# Current clinical practice of prurigo nodularis in Japan: A cross‐sectional web‐survey among dermatologists

**DOI:** 10.1111/1346-8138.17400

**Published:** 2024-09-03

**Authors:** Hiroyuki Murota, Mai Matsumoto, Kazuhiko Arima, Takuo Yoshida, Hiroyuki Fujita

**Affiliations:** ^1^ Department of Dermatology Nagasaki University Nagasaki Nagasaki Japan; ^2^ Sanofi, Tokyo Opera City Tower Tokyo Japan

**Keywords:** dermatology, disease severity, Japan, prurigo, pruritus

## Abstract

Prurigo nodularis (PN) is a chronic inflammatory skin disease associated with intense pruritic nodules. The unclear patho‐etiological mechanisms of PN cause difficulty in disease management; and there is a paucity of information on the current diagnosis and treatment options for PN in Japan. To describe the current management from a dermatologists' perspective we conducted a web‐based survey (UMIN Clinical Trial Registry UMIN000047643) in 2022 among dermatologists from a Japanese commercially available physician panel, who had seen at least one patient with PN within the last 3 months. The survey included 117 dermatologists. The dermatologists diagnosed PN mainly by confirming clinical signs and patient interviews, while to assess the severity of PN, the number of pruritic nodules and the degree of itching were primarily utilized. Topical corticosteroids and antihistamines were the most used drugs, as recommended in the current guidelines on the diagnosis and treatment of prurigo. Dermatologists' treatment satisfaction decreased with increasing assumed severity of PN; almost 65% dermatologists were not satisfied with the treatment of severe PN. These results suggest the need of more effective medications and diagnostic tools for better management of PN in Japan.

## 1 INTRODUCTION

Prurigo nodularis (PN) is a chronic, inflammatory skin disease associated with intense itch that has a greater impact on patients’ quality of life (QOL) than other skin diseases.^1^, ^2^ Various components of the cutaneous, immune, and nervous systems play roles in the pathogenesis of PN.^3^, ^4^ The classification and concept of PN remain ill‐defined, while the diagnosis, assessment, and management of the disease are still insufficiently explored. To understand the perspectives of dermatologists on current treatments of PN in Japan, we conducted a web‐based survey among dermatologists who treat patients with PN.

## 2 METHODS

### 2.1 Study design

A web‐based survey was conducted between April and June 2022 in Japan (UMIN‐Clinical Trials Registry UMIN000047643) among dermatologists and patients with PN. Following the report on the patient cohort,^5^ the present report presents findings from the dermatologist cohort. Dermatologists, who had seen at least one patient with PN in the past 3 months, from a physician panel provided by PLAMED, Inc (Tokyo, Japan), answered online questionnaires on the disease. The questions concerned assessing attributes of the practice setting (either hospital‐based or clinic‐based), diagnostic measures, treatment details, treatment satisfaction, and their perception of patient disease burden. All participants provided informed consent to publication of the results.

## 3 RESULTS

### 3.1 Demographics of participants

The analysis cohort included 117 dermatologists among whom 109 (93.2%) were board‐certified by the Japanese Dermatological Association (JDA) (Table 1). In total, 61.5% respondents worked in hospitals, and the average number of patients with PN treated in the past 3 months was 15.3.

**TABLE 1:** Participant demographics.

Item		Overall	Dermatologists in clinic (CL)	Dermatologists in hospital (HP)
No. of participating dermatologists (No. of JDA‐certified dermatologists)		117 (109)	45 (42)	72 (67)
Age *n* (%), years	≤39	24 (20.5)	1 (2.2)	23 (31.9)
	40–49	39 (33.3)	16 (35.6)	23 (31.9)
	50–59	31 (26.5)	15 (33.3)	16 (22.2)
	60 ≤	23 (19.7)	13 (28.9)	10 (13.9)
Number of PN patients in last 3 months, median	Total	15.3	16.5	14.6
	Mild	4.4	5.4	3.8
	Moderate	6.7	7.0	6.5
	Severe	2.3	1.5	2.9

*Note*: Healthcare facility: Clinic (CL): facility with 0–19 beds; Hospital (HP): facility with 20 beds or more.

### 3.2 Diagnostic measures

**FIGURE 1:**
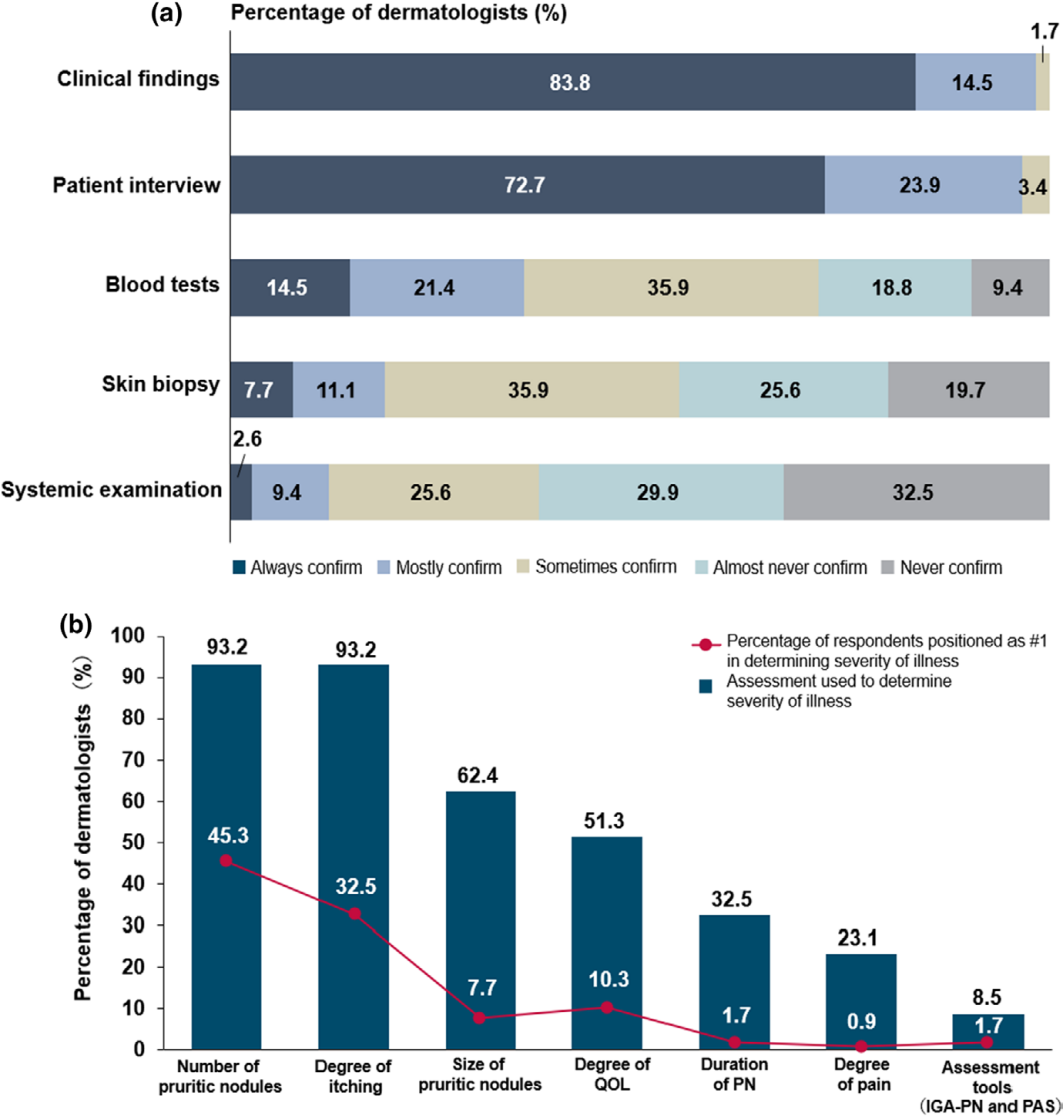
(a) Overall diagnostic modality performed to confirm the diagnosis of prurigo nodularis (PN). (b) Overall diagnostic modality assessing the disease severity of PN. IGA, Investigator's Global Assessment; PAS, Prurigo Active Score; PN, prurigo nodularis; QOL, quality of life.

A definite diagnosis of PN was based mainly on clinical findings and patient interviews. For these two items, all the dermatologists responded answered either “always”, “mostly”, or “sometimes” confirm. In contrast, blood test, skin biopsy, and systemic examination for routine confirmation were less used by 71.8%, 54.7%, and 37.6% of respondents, respectively (either “sometimes/always/mostly” confirm; Figure 1a). Compared to dermatologists in clinics, more dermatologists in hospitals performed blood tests (19.4% vs 6.7%) and skin biopsies (11.1% vs 2.2%) (Figure S1).

### 3.3 Assessment of PN severity

In the absence of descriptions about the severity classification for PN in the practice guidelines,^6^ the classification in this survey was based on the physicians' perception. Dermatologists used the “number of pruritic nodules” and “degree of itching” most frequently to assess the PN severity (93.2% each), followed by “size of pruritic nodules” (62.4%), “degree of QOL” (51.3%), “duration of PN” (32.5%), and “degree of pain” (23.1%). Only a few dermatologists (8.5%) used established assessment tools such as the Investigator's Global Assessment‐PN or Prurigo Activity Score^7^ (Figure 1b). Dermatologists working in hospitals ranked “number of pruritic nodules” first (50.0%) and “degree of itching” second (29.2%) as the most important aspect for severity assessment, while those working in clinics ranked “number of pruritic nodules” (37.8%) less and “degree of itching” (37.8%) more (Figure S2). Dermatologists in clinics tended more to assess patient‐reported outcomes along with major disease symptoms, while those in hospitals more commonly assessed etiological parameters as well as number and size of PN.

### 3.4 Current treatment and treatment satisfaction of dermatologists

**FIGURE 2:**
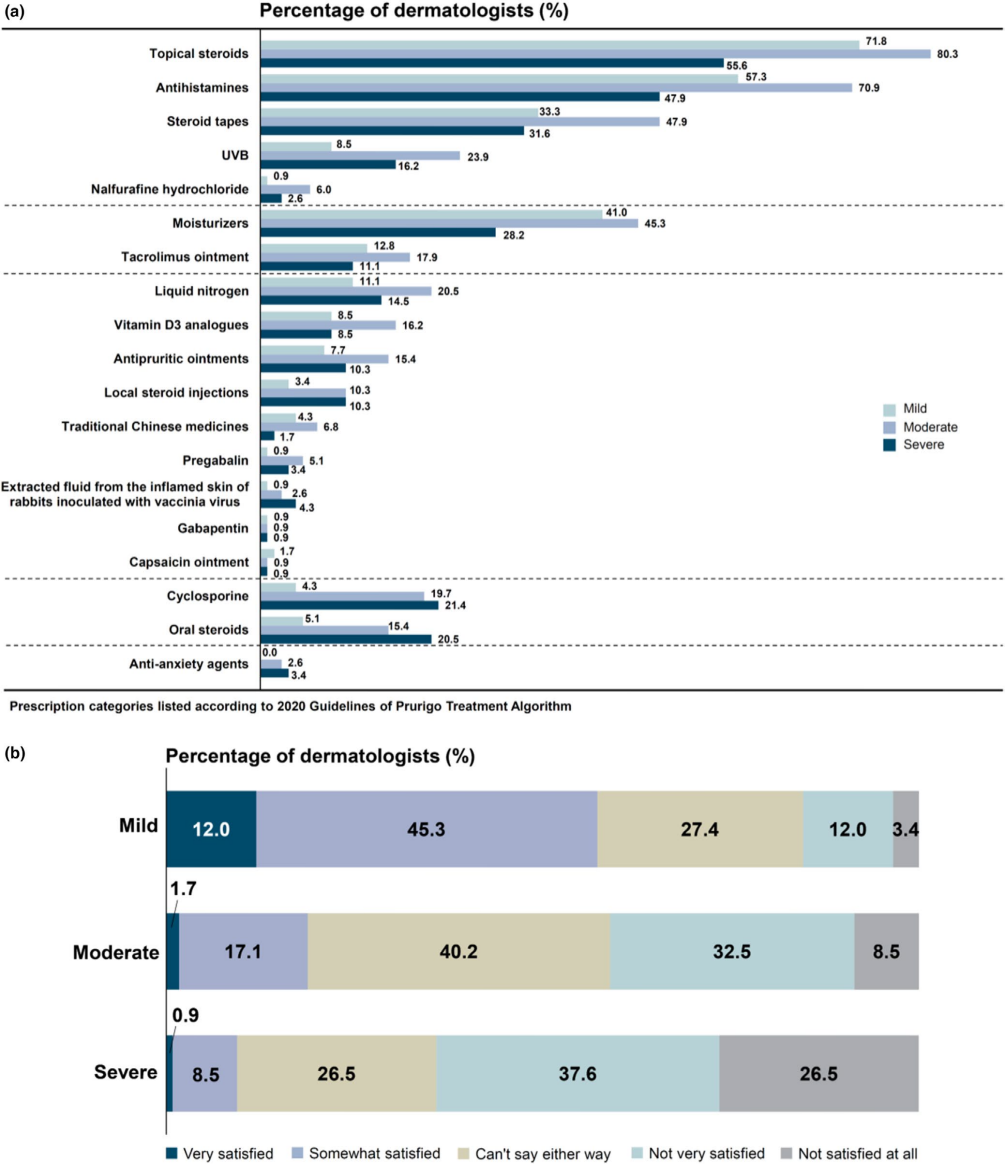
(a) Drugs used to treat prurigo nodularis (PN) according to disease severity. (b) Satisfaction among dermatologists with PN treatment according to disease severity. UVB, ultraviolet B.

The practical guidelines for prurigo describe several treatment options.^7^, ^8^ In the present survey, topical corticosteroids (TCS) (71.8%, 80.3%, and 55.6% for mild, moderate, and severe disease, respectively) and antihistamines (57.3%, 70.9%, and 47.9% for mild, moderate, and severe disease, respectively) were most used, as expected.^6^ Overall, 21.4% and 20.5% of dermatologists reported using cyclosporine or oral corticosteroids (OCS), respectively, within the past 3 months for treating patients with severe disease (Figure 2a).

For dermatologists in both clinics and hospitals, TCS and antihistamines were the most used medications (Figure S3). Other treatment agents, including off‐label drugs, were also used, particularly for those with moderate or severe disease. The most common reason for patients with mild disease to stop coming to see the physician was “symptoms improved” (68.4%). However, with disease severity of moderate and above, the percentage for “symptoms improved” decreased while “symptoms remained unaltered” and “symptoms deteriorated” increased considerably (Figure S4).

Most dermatologists were either “very” or “somewhat” satisfied with outcomes obtained from treating mild disease (57.3%), while only 18.8% and 9.4% were satisfied with outcomes obtained in moderate and severe disease, respectively (Figure 2b). In contrast, 15.4%, 41.0%, and 64.1% of dermatologists were either “dissatisfied” or “not very satisfied” with the treatment of patients with mild, moderate, and severe diseases, respectively (Figure 2b). These trends were similar among dermatologists in hospitals and clinics, although a few dermatologists in hospitals were “very” satisfied with the current treatment (Figure S5). Additionally, 34.2% and 6.8% of dermatologists stated that for mild and moderate PN, respectively, treatment could be ended within 6 months. Regarding the time to the complete cure of PN, 36.8% of dermatologists responded more than 1 year for mild disease while 94.0% and 64.1% responded more than 1 year and 3 years, respectively, for severe disease (Figure S6).

## 4 DISCUSSION

Despite recent advances in understanding the pathophysiology of PN, diagnosis and severity assessment of PN are still based mainly on clinical findings and patient interviews. Although patients with PN have a wide range of symptoms and severities, the number of nodules and degree of itching remain the most used parameters to assess disease severity. With a disease burden as diverse as atopic dermatitis (AD),^5^ severity of PN should be assessed in future using the core outcome set of signs, symptoms, long‐term control, and QOL, as recommended for AD.^8^ Patient‐reported outcome tools for disease control as in AD^9^, ^10^ and well‐validated biomarkers for better diagnosis and outcome measurement are warranted.

A cross‐sectional study conducted in 15 centers in 12 European countries reported a lack of consensus on diagnosis and management of PN among the dermatologists.^11^ We observed a few differences in the practice approaches favored by dermatologists working in clinics and hospitals. Unclear pathophysiology, diverse symptom manifestation of PN, and a lack of universality in the concept and therapeutic norms impede the treatment of PN.^11^ Our survey revealed that Japanese dermatologists tended to adopt various treatment options, including off‐label drugs, following recommendations made in the International Forum for Study of Itch and the JDA prurigo guidelines.^6^, ^12^ TCS were used infrequently for patients with severe PN in comparison to those with mild and moderate PN. This scenario might reflect the possibility of patients' and physicians' preference for systemic therapies such as OCS or cyclosporine over topical therapies during the prolonged and refractory treatment course of severe PN. Having said that, there are a few studies conducted in other countries which reported the use of therapeutics based on the severity of PN. While a few studies report proportions of prescribed therapies by severity of PN, the use of TCS in Germany and the United States seemed to be less than in Japan.^13^, ^14^ The use of OCS in Japan for treating PN was comparable to that in the United States, but was more frequent than in Germany.^13^ The frequency of use of anti‐anxiety agents and antidepressants was high in Germany (ca. 20%).^13^ The need to delineate standardized diagnostic and treatment algorithms for PN is progressively gaining relevance.^15^

The present survey had a few limitations. The respondents in this survey may not represent all dermatologists treating patients with PN in Japan. We tried to mitigate this potential bias by confirming that a high proportion of participants were certified, well‐trained dermatologists. A possibility of recall bias, nevertheless, exists as the data were self‐reported by the dermatologists.

In conclusion, this is the first web‐based survey of Japanese dermatologists to assess the current diagnosis and treatment of PN. The findings confirmed that dermatologists usually rely on numbers of nodules and degree of itch for severity assessment. Dermatologists are generally not satisfied with the treatment for patients with severe PN which leads to the use of off‐label drugs listed in the guidelines. More effective treatment options, especially for severe patients, are needed for better management of patients with PN.

## CONFLICT OF INTEREST STATEMENT

Hiroyuki Murota received honorarium for lectures from AbbVie, Eli Lilly, Maruho, and Sanofi. Kazuhiko Arima, Takuo Yoshida, and Hiroyuki Fujita are employees of, and stockholders of Sanofi K.K. Mai Matsumoto has no perceived conflict of interest. Sanofi K.K. funded the study and medical writing support.

## ETHICS STATEMENT

The study was approved by the Ethics Review Board of Medical Corporation Tohkei‐kai Kitamachi Clinic, Tokyo, Japan, on March 16, 2022.

## Supporting information


Data S1.
