# Supplementary material for: Current clinical practice of prurigo nodularis in Japan: A cross‐sectional web‐survey among dermatologists
Source: J Dermatol. 2024 Sep 3;52(2):348–52. doi: 10.1111/1346-8138.17400 (PMC11807362; doi:10.1111/1346-8138.17400)
Supplement: Supplementary file 1 — Data S1. [file JDE-52-348-s001.docx]

**SUPPORTING INFORMATION**


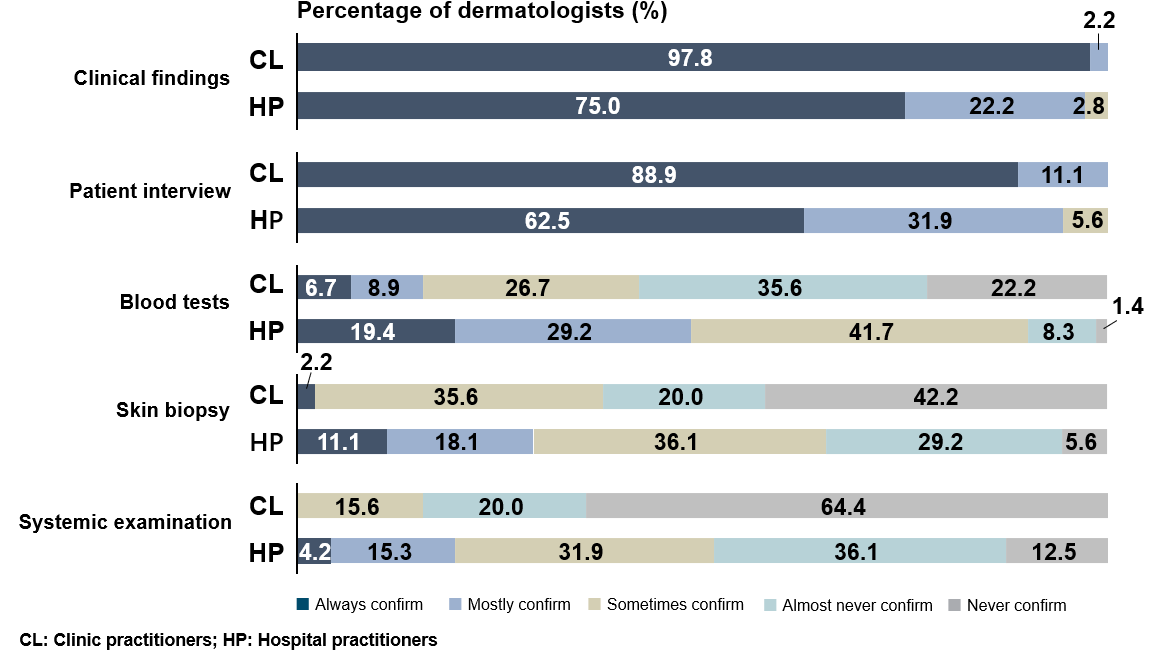
Supplementary Figure S1: Tests performed to confirm the diagnosis of PN in hospitals and clinics.


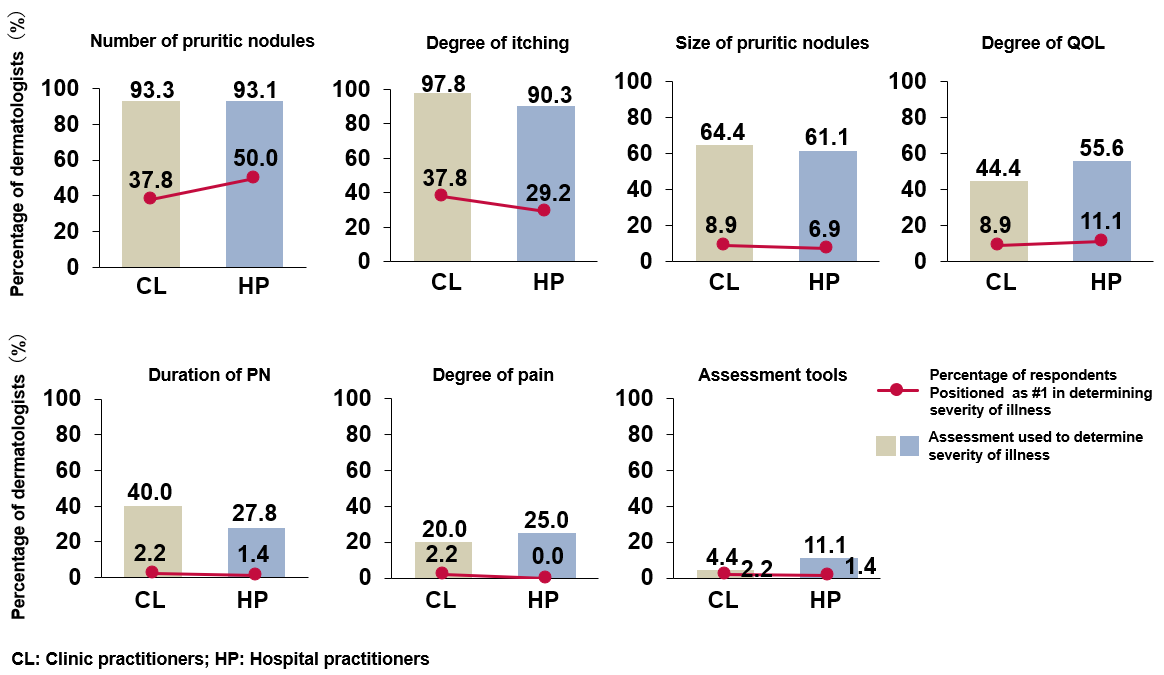


Supplementary Figure S2: Criteria for clinical assessment of degree of severity of prurigo nodularis adopted by dermatologists in clinic (CL) and dermatologists in hospital (HP). PN, Prurigo nodularis; QOL, quality of life.

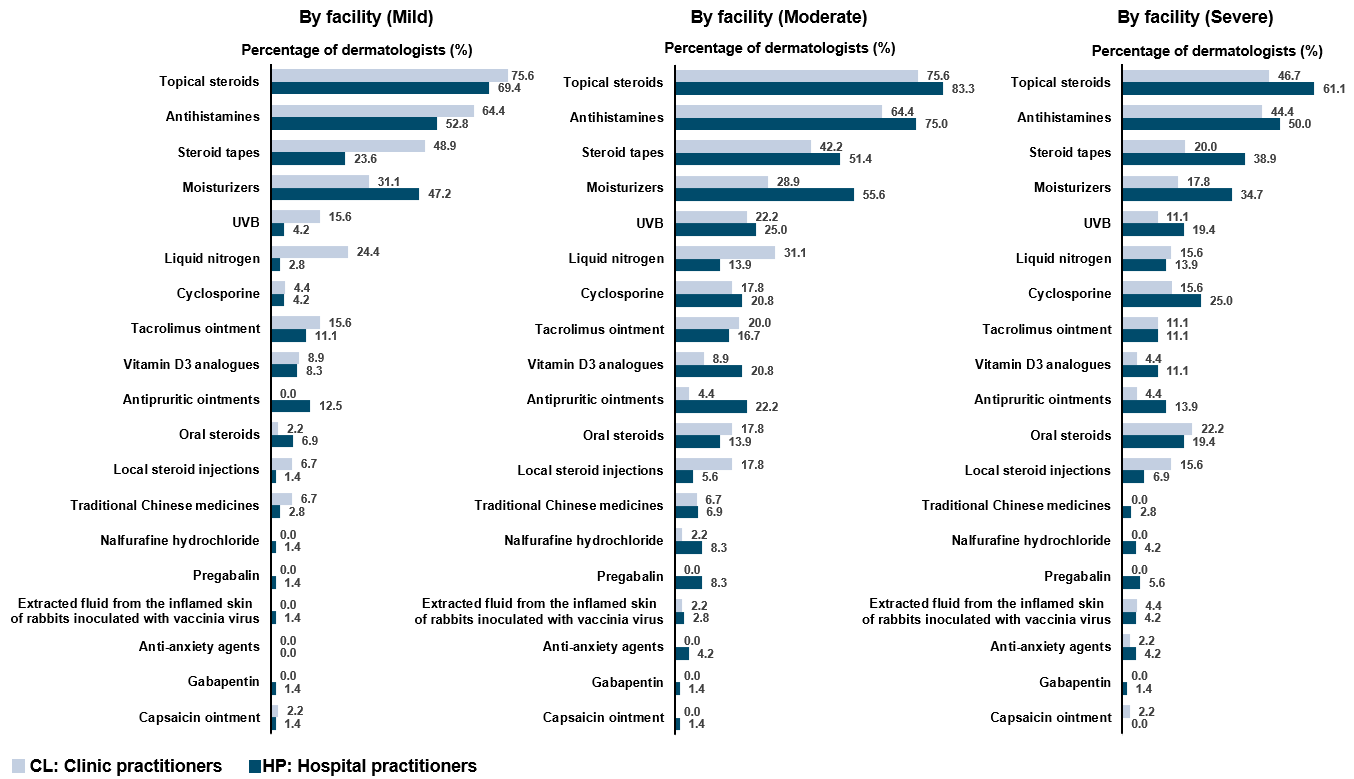

Supplementary Figure S3: Drugs used to treat prurigo nodularis according to disease severity in hospital and clinic facilities. UVB, ultraviolet B.


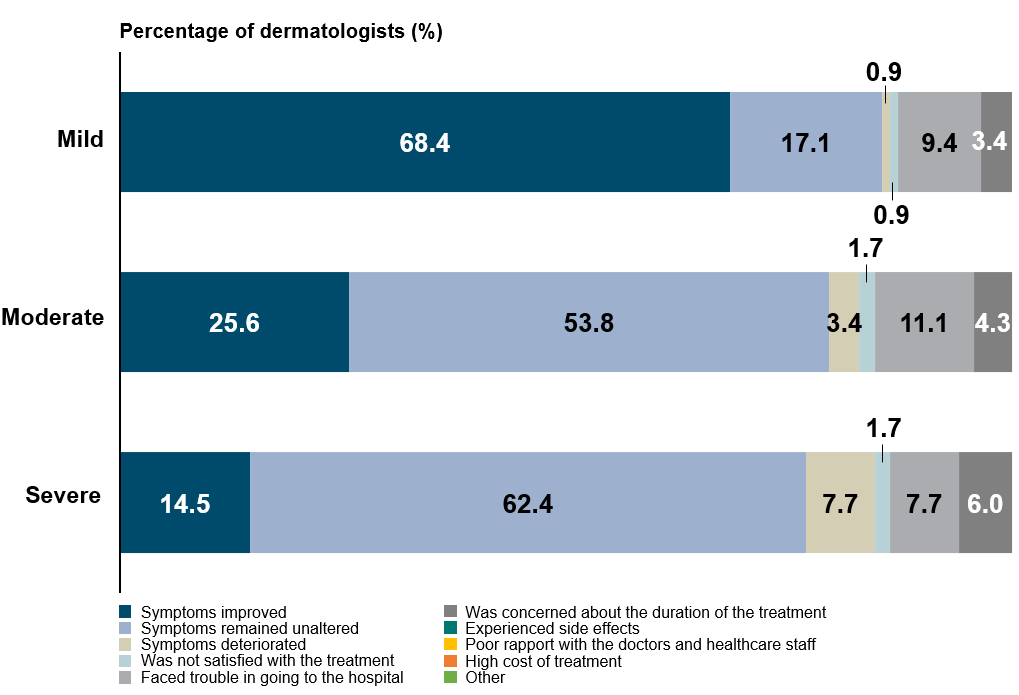


Supplementary Figure S4: Dermatologist’s perception on reasons for patients to stop coming to the hospital.


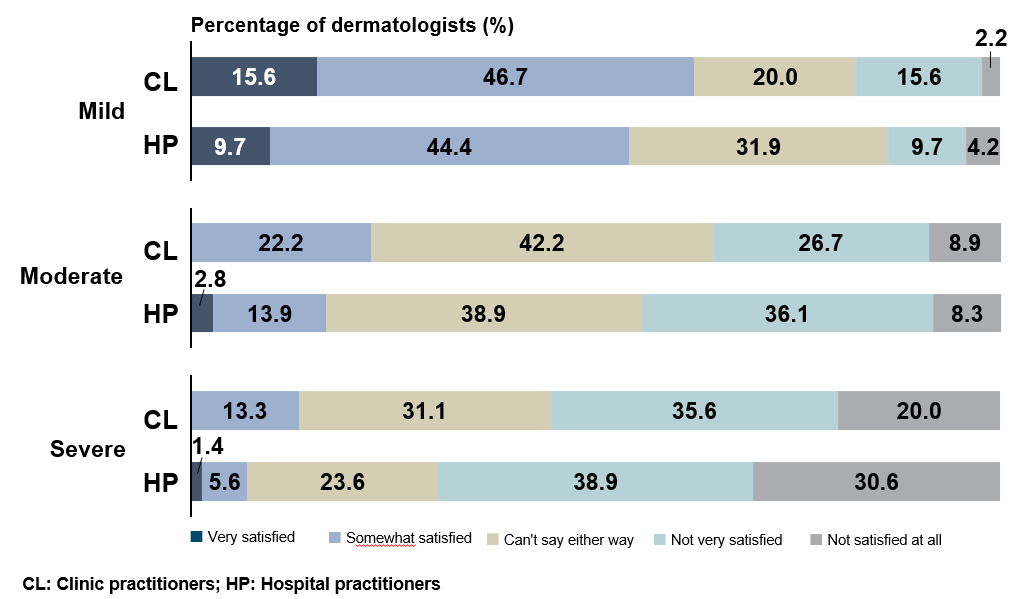
Supplementary Figure S5: Satisfaction among dermatologists in clinic (CL) and dermatologists in hospital (HP) with prurigo nodularis treatment of varying disease severity.


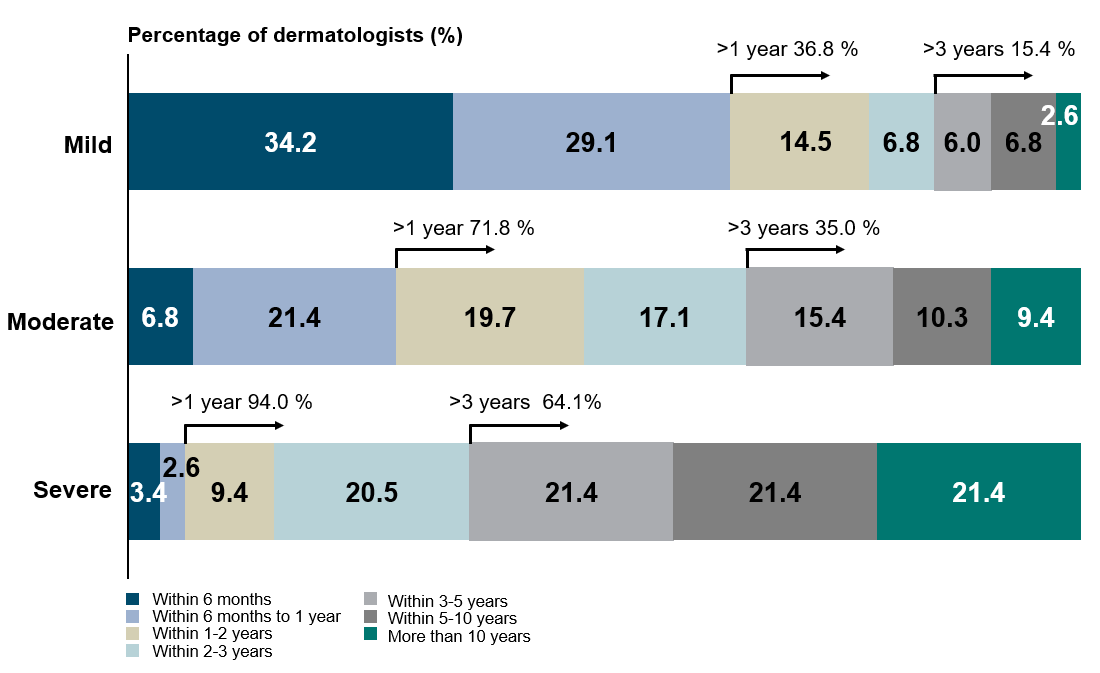


Supplementary Figure S6: Dermatologist’s perception on time to complete prurigo nodularis treatment of varying disease severity.
